# When Home Is Not a Safe Place: Confronting Domestic Violence During the COVID-19 Era

**DOI:** 10.31661/gmj.v12i.2814

**Published:** 2023-04-25

**Authors:** Nader Aghakhani, Mohammad Delirrad, Elham Jafari

**Affiliations:** ^1^ Food and Beverages Safety Research Center, Urmia University of Medical Sciences, Urmia, Iran; ^2^ Department of Forensic Medicine, School of Medicine, Food and Beverages Safety Research Center, Taleghani Hospital, Imam Khomeini Hospital, Urmia University of Medical Sciences, Urmia, Iran

**Keywords:** Domestic Violence, COVID-19

## Dear Editor,

Domestic violence refers to a variety of physical, economic, psychological, or potentially sexual intimate partner abuse within a familiar environment. It can be known as a public health issue with serious implications, and a violation of human rights [1].

When the worldwide coronavirus disease 2019 (COVID-19) pandemic increases, many countries are taking enthusiastic programs, such as encouraging individuals to adopt social distance, limitation of business and schools, and restriction of travel. Unfortunately, these programs can result in a reduction of optimal safety [2].

During the COVID-19 crisis, health professionals were at the forefront of fighting against disaster. Therefore, it is crucial to think of a safe condition for the victims to declare and do something against invasive behaviors. One approach is to ask them if they feel safe in a fair and safe manner. However, it is critical that health professionals have the opportunity and motivation to listen to and respond to the commonly useful ways in which victims demonstrate that they are at risk of a dangerous condition [3].

For providing advice and counselling, the use of online technologies is necessary for the victims, who may not have access to these abilities. This emphasizes the importance of providing various technological types of support and recognizes that people may be unable to seek help or enough care while social obstacles affect their safety, and well-being. Additionally, it is crucial to organize educational groups and community awareness missions to take action against unsafe opinions and habits [4].

Consultants, therapists, advocates, and helpline practitioners working in rehabilitation services should provide adequate support and care to victims and survivors exposed to imminent risks during the COVID-19 pandemic, and should incorporate disaster readiness into upcoming service delivery procedures, building on previous lessons with a complete understanding of the psychological consequences of social isolation on survivors and the abuse tactics of perpetrators, and developing urgent strategies in creating, testing, and mapping out digital and digitally delivered responses. On the other hand, governments should reinforce these services to remain open, access to personal protective equipment to help their own clients [5].

The occurrence of domestic violence has grown during the COVID-19 pandemic. Accordingly, it is suggested to increase knowledge about victimization rates and reports in order to make the appropriate referral and reduce the burden of the problem. Also, the information services, such as telehealth, hotlines, and support and counseling centers, should be accessible via social and traditional or established broadcasting or publishing media, as well as effective health care settings.

## Conflict of Interest

The authors declare no conflict of interests.
